# *Cryptococcus gattii* Induces a Cytokine Pattern That Is Distinct from Other Cryptococcal Species

**DOI:** 10.1371/journal.pone.0055579

**Published:** 2013-01-31

**Authors:** Teske Schoffelen, Maria-Teresa Illnait-Zaragozi, Leo A. B. Joosten, Mihai G. Netea, Teun Boekhout, Jacques F. Meis, Tom Sprong

**Affiliations:** 1 Department of Medicine and Nijmegen Institute for Infection, Inflammation & Immunity (N4i), Radboud University Nijmegen Medical Centre, Nijmegen, The Netherlands; 2 Department of Bacteriology and Mycology, Instituto Pedro Kourí, Havana, Cuba; 3 CBS-KNAW Fungal Biodiversity Centre, Utrecht, The Netherlands; 4 Shanghai Key Laboratory for Molecular Medical Mycology, Changzheng Hospital, Second Military Medical University, Shanghai, China; 5 Department of Medical Microbiology and Infectious Diseases, Canisius-Wilhelmina Hospital, Nijmegen, The Netherlands; 6 Department of Medicine, Canisius-Wilhelmina Hospital, Nijmegen, The Netherlands; CNRS, France

## Abstract

Understanding more about the host's immune response to different *Cryptococcus* spp. will provide additional insight into the pathogenesis of cryptocococcis. We hypothesized that the ability of *C. gattii* to cause disease in immunocompetent humans depends on a distinct innate cytokine response of the host to this emerging pathogen. In the current study we assessed the cytokine profile of human peripheral blood mononuclear cells (PBMCs) of healthy individuals, after *in vitro* stimulation with 40 different well-defined heat-killed isolates of *C. gattii*, *C. neoformans* and several hybrid strains. In addition, we investigated the involvement of TLR2, TLR4 and TLR9 in the pro-inflammatory cytokine response to *C. gattii*. Isolates of *C. gattii* induced higher concentrations of the pro-inflammatory cytokines IL-1β, TNF-α and IL-6 and the Th17/22 cytokine IL-17 and IL-22 compared to *C. neoformans* var *neoformans* and *C. neoformans* var *grubii*. In addition, clinical *C. gattii* isolates induced higher amounts of cytokines than environmental isolates. This difference was not observed in *C. neoformans* var. *grubii* isolates. Furthermore, we demonstrated a likely contribution of TLR4 and TLR9, but no role for TLR2, in the host's cytokine response to *C. gattii*. In conclusion, clinical heat-killed *C. gattii* isolates induced a more pronounced inflammatory response compared to other *Cryptococcus* species and non-clinical *C. gattii*. This is dependent on TLR4 and TLR9 as cellular receptors.

## Introduction

The incidence of cryptococcosis has increased dramatically over the past decades, due in a large part to the global HIV pandemic. More than 600,000 deaths are estimated to occur each year as a result of cryptococcal meningoencephalitis [1]. The species *C. neoformans* is an opportunistic pathogen mainly affecting immunocompromised hosts. In contrast, *C. gattii* mainly causes disease in apparently immunocompetent hosts at lower incidence [2], [3]. *C. gattii* is emerging over the past decade as a pathogen in the Pacific North-West of North America and has caused a large outbreak on Vancouver Island [4], [5]. This outbreak was mainly caused by a single, hypervirulent genotype of *C. gattii*, namely AFLP6A/VGIIa [6].

Cells of the innate immune system are important for initial defense against pathogens. Upon contact with pathogens, they produce pro-inflammatory cytokines such as tumor necrosis factor (TNF)-α, Interleukin (IL)-1β and IL-6, thereby initiating a specific adaptive cellular immune response. Anti-inflammatory cytokines such as IL-1RA are also produced and act as downregulators of this immune response. Of particular interest for fungal infections, the cytokines IL-1β and IL-6 in the presence of IL-23 induce the development of T-helper (Th)17 cells. IL-17 and IL-22, the major cytokines excreted by Th17 cells, have several pro-inflammatory functions, one of which is eliciting defensin production by epithelial cells [7]. Previous studies have shown a crucial role of Th17 cells in human antifungal defense against mucosal *Candida albicans* infections [8]–[10]; but the role of this particular Th-lymphocyte subset in anti-cryptococcal defense is not clear.

Which cytokines are released depends on recognition of microbial components by pattern recognition receptors (PRRs) on the cells of the innate immune system. Toll-like receptors (TLRs), a well-defined set of PRRs, are expressed on a variety of cells and are important mediators of pro-inflammatory cytokine release. However, their role in mediating cytokine response to *Cryptococcus* spp. is being debated [11]–[15].

Understanding more about the host's immune response to different *Cryptococcus* spp, will provide additional insight into the pathogenesis of cryptocococcis. We hypothesized that the ability of *C. gattii* to cause disease in immunocompetent humans depends on a distinct innate cytokine response of the host to this emerging pathogen. Therefore, in the current study we assessed the cytokine profile of human peripheral blood mononuclear cells (PBMCs) of healthy individuals, after *in vitro* stimulation with well-defined heat-killed isolates of *C. gattii*, *C. neoformans* and several hybrids. In addition, we investigated the involvement of TLR2, TLR4 and TLR9 in the pro-inflammatory cytokine response to *C. gattii*.

## Results

### Quantitative comparison of cytokine induction between different *Cryptococcus* spp

**Figure 1:**
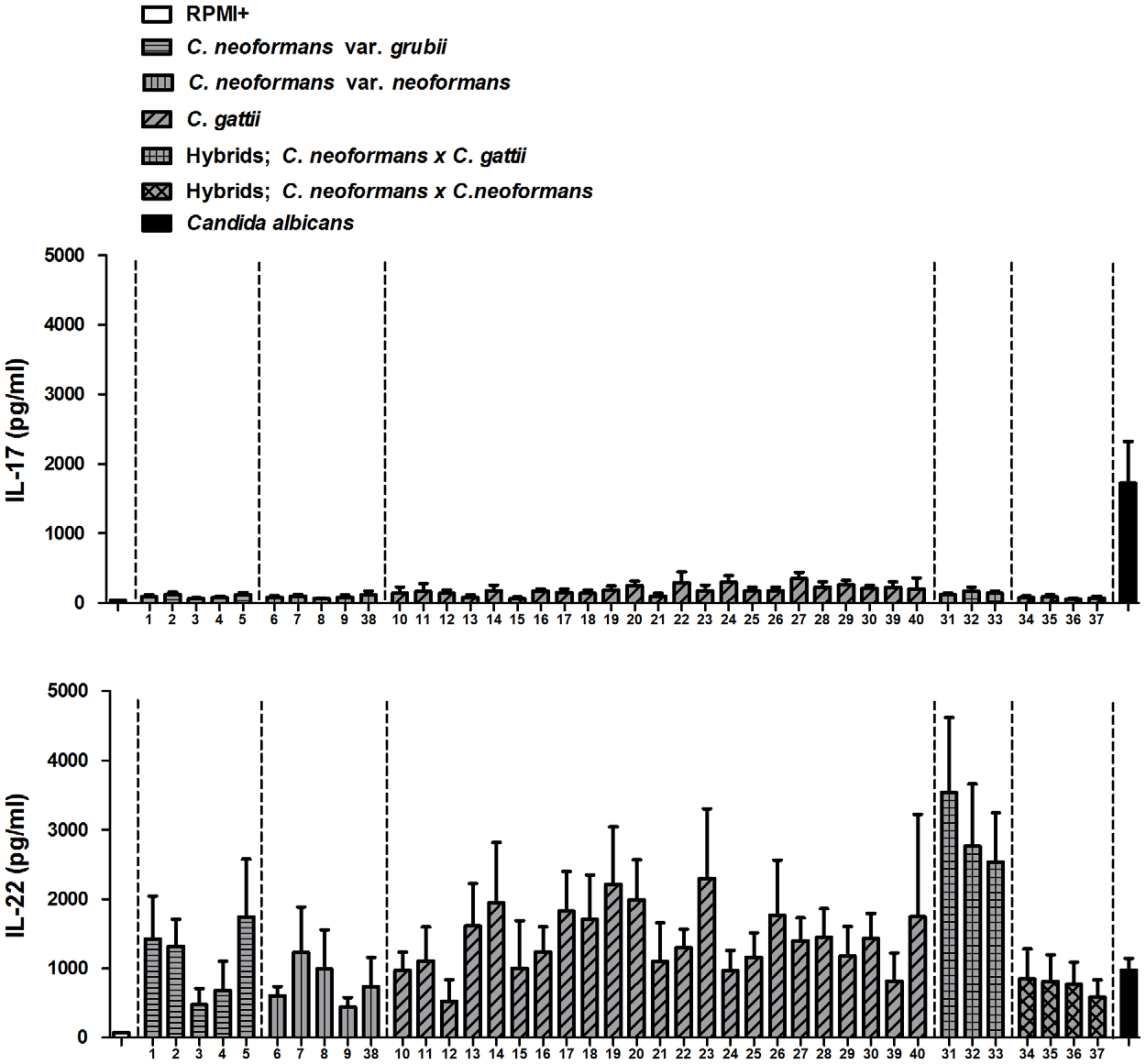
All forty *Cryptococcus* strains induce low amounts of IL-17, but high amounts of IL-22. IL-17 and IL-22 production after 7 d by PBMCs stimulated with RPMI+, either one of 40 different heat-killed *Cryptococcus* strains [10^7^ microorganisms/mL] or heat-killed *Candida albicans* [10^5^ microorganisms/mL] is shown respectively. Mean values ± SE (n = 5) of three independent experiments are presented.

We determined the concentration of several cytokines produced by PBMCs upon stimulation with 40 different heat-killed *Cryptococcus* species complex isolates in order to elucidate the cytokine milieu in cryptococcal infection and to explore differences between the species. In preliminary experiments, we determined that the minimal concentration of yeasts necessary to induce cytokine production is 10^7^ microorganisms/mL (data not shown). There was substantial inter-strain variation in the production of the pro-inflammatory cytokines IL-1β, TNF-α, IL-6 and the anti-inflammatory cytokine IL-1Ra. TNF-α and IL-1β were induced in low amounts (up to 300 pg/mL). Interestingly, production of these cytokines using a 100-fold lower concentration of *Candida albicans* was much higher (data not shown). Results for the induction of T-cell derived cytokines IL-17 and IL-22 after 7 days of incubation are shown in Figure 1. It appeared that the studied *Cryptococcus* strains induce low amounts of IL-17 but substantial quantities of IL-22, again with significant inter-strain variation in the production of these cytokines.

**Figure 2:**
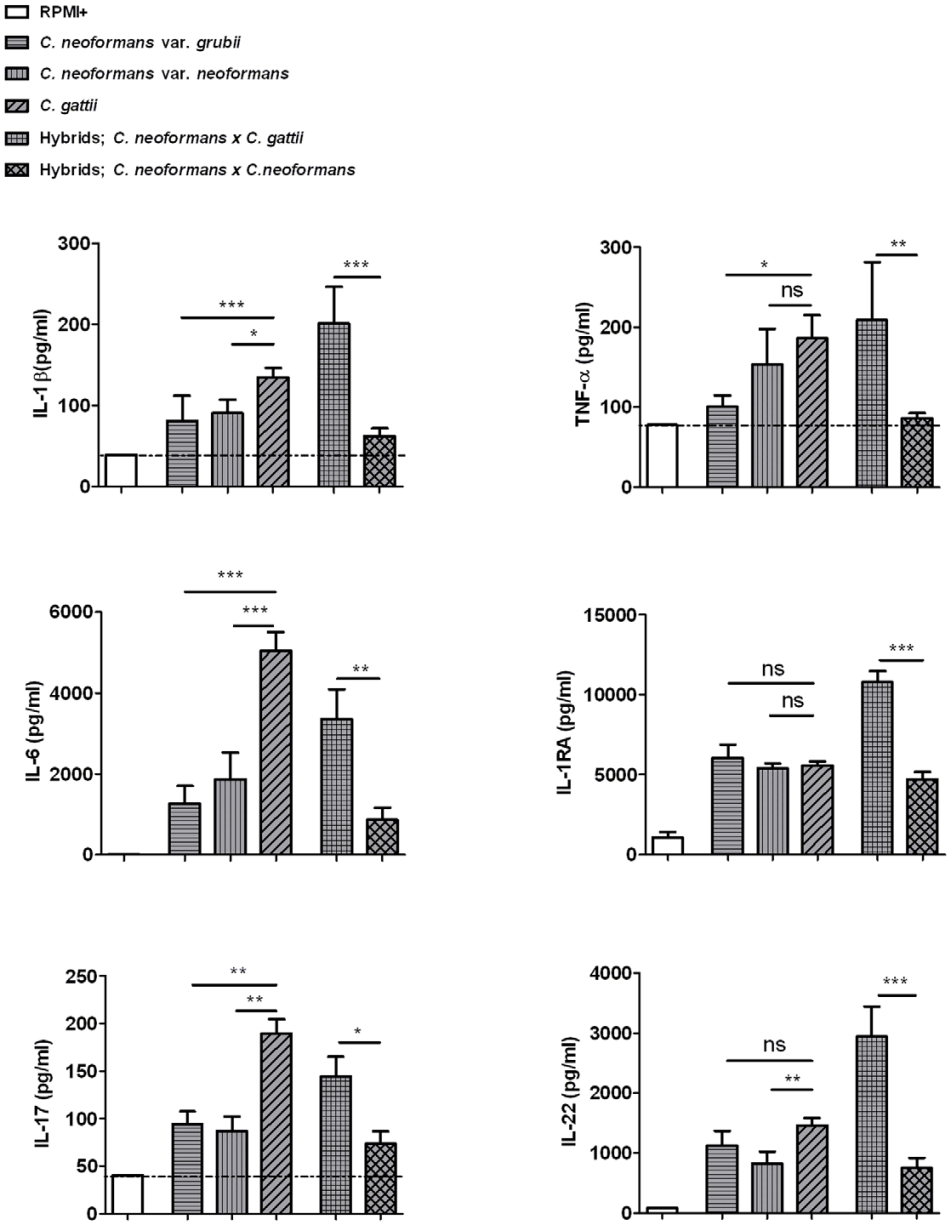
Comparison of *C. gattii* isolates and interspecies hybrids with *C. neoformans* isolates and hybrids between both *C. neoformans* varieties. The forty heat-killed *Cryptococcus* isolates are grouped according to (sub)species. Cytokine production by human PBMCs after 24 h (IL-1β, TNF-α, IL-6 and IL-1Ra) and 7 d (IL-17 and IL-22) incubation is shown. Mean values (n = 5 to 7) ± SE of three independent experiments are presented. *, p 0.01 to 0.05; **, p 0.001 to 0.01; ***, p<0.001. The horizontal line represents the lower detection limit.

Figure 2 shows a quantitative comparison of cytokine induction between two varieties of *C. neoformans*, *C. gattii* and various hybrid isolates. *C. gattii* was a more potent inducer of the pro-inflammatory cytokines TNF-α, IL-1β, IL-6 and the T-cell cytokines IL-17 and IL-22, compared to both *C. neoformans* varieties. The different species did not differ with regard to IL-1Ra induction. Interestingly, the interspecies hybrids containing *C. gattii* as a partner of the mating pair induced significantly higher cytokine production than hybrids which were the result of mating between the two varieties of *C. neoformans*. This suggests that an inheritable factor is responsible for the difference in cytokine production.

### Quantitative comparison of cytokine induction between environmental and clinical strains within the *Cryptococcus* species complex

**Figure 3:**
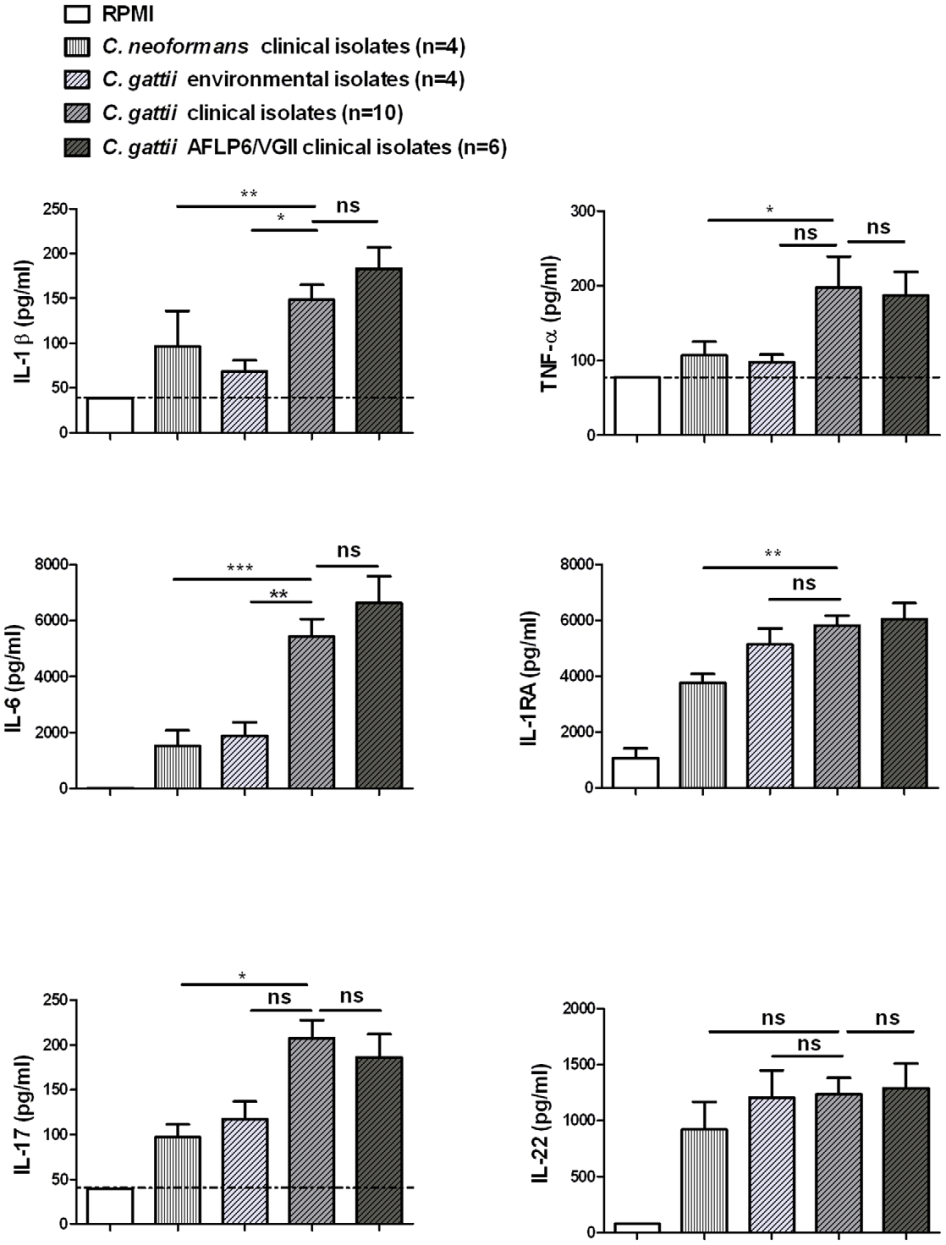
Comparison of cytokine production by PBMCs induced by clinical or environmental cryptococcal isolates. Heat killed clinical isolates of *C. gattii* are compared to environmental *C. gattii* isolates and to clinical isolates of *C. neoformans*. The clinical isolates of *C. gattii* genotype AFLP6/VGII are depicted separately. Mean values (n = 5 to 7) ± SE values of three independent experiments are presented. *, p 0.01 to 0.05; **, p 0.001 to 0.01; ***, p<0.001. The horizontal line represents the lower detection limit.

Sixteen clinical *C. gattii* isolates (isolates 10,12,14,18,19–21,23–29,39,40), of which six isolates belonging to the genotype AFLP6/VGII which was involved in the Vancouver Island outbreak, were compared to four environmental *C. gattii* isolates (isolates 13,15,16,17), as well as to four clinical *C. neoformans* isolates (isolates 1,4,5,9), with regard to the cytokine induction (Figure 3). Clinical *C. gattii* isolates induced significantly higher IL-1β and IL-6 amounts compared to environmental isolates. Moreover, clinical *C. gattii* isolates also induced higher IL-1β, IL-6, TNF-α, IL-1Ra and IL-17 than clinical *C. neoformans* isolates. The *C. gattii* genotype AFLP6/VGII, however, induced no higher amounts of other cytokines compared to the other clinical *C. gattii* isolates.

**Figure 4:**
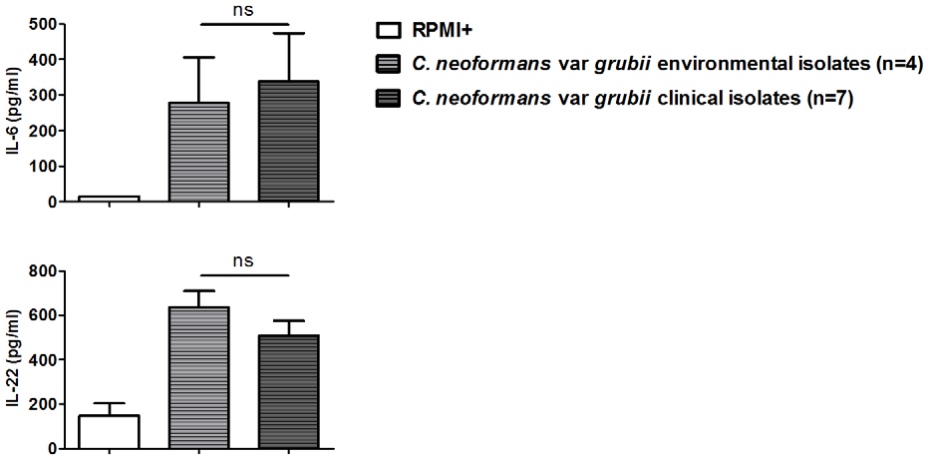
Comparison of cytokine production by PBMCs induced by clinical or environmental *C. neoformans* var *grubii* isolates. Cytokine production by human PBMCs after 24 h (IL-6) and 7 d (IL-22) incubation with heat-killed isolates is shown. Mean values (n = 7) ± SE of three independent experiments are presented. ns, not significant.

In a different panel of Cuban *C. neoformans* var *grubii* isolates, comparison of clinical with environmental isolates showed no significant difference (*P* value for IL-6 and IL-22: 0.19 and 0.07 respectively) in cytokine production (Figure 4). The induction of low levels of cytokines by *C. neoformans* var *grubii* isolates, as seen in the panel of 40 isolates, was confirmed.

### Involvement of different Pattern Recognition Receptors (PRRs) in cytokine production induced by *C. gattii*

**Figure 5:**
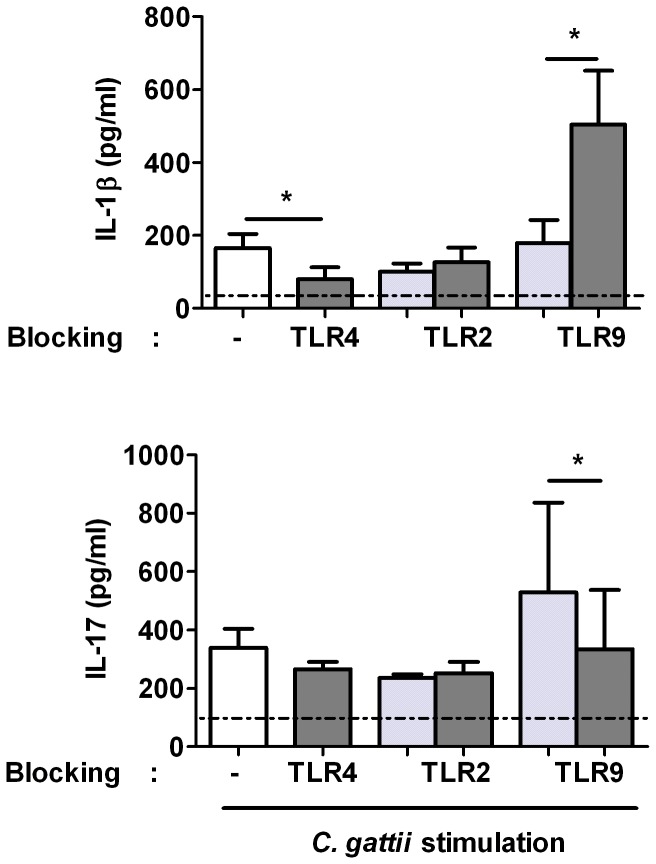
The role of TLR2, TLR4 and TLR9 in IL-1β and IL-17 induction by *C. gattii*. Cytokine production by human PBMCs preincubated for one hour with culture medium (white bar) or PRR blocking reagents (dark gray bars) or their control (light gray bar) prior to stimulation with heat-killed *C. gattii* (strain B5742) [10^7^/ml]. IL-1β is determined after 24 h incubation, IL-17 is determined after 7 d incubation. Mean values ± SE of eight individuals in 4 independent experiments (IL-17) or six individuals in 5 independent experiments (IL-1β) (with exclusion of additional four individuals with undetectable cytokine induction by *C. gattii*) are presented. *, p 0.01 to 0.05. The horizontal line represents the lower detection limit.

To assess which PRRs are involved in recognizing *C. gattii,* we performed experiments in which PBMCs were preincubated for one hour with specific PRR blocking reagents prior to stimulation with heat-killed *C. gattii* or, as a control, culture medium. Stimulation with culture medium showed undetectable levels for all cytokines (not shown). Blocking TLR2 had no effect on cytokine production by *C. gattii*, whereas this antibody significantly inhibited IL-1beta production after stimulation with Pam3cys (a known TLR-2 ligand) (Figure S1). Blocking TLR4 significantly diminished IL-1β induction by *C. gattii*, with a trend towards significance for TNF-α (*P* = 0.06). Interestingly, blocking TLR9 led to significantly higher concentrations of IL-1β induced by *C. gattii* compared to its control, and a trend towards significance (*P* = 0.06) was found for TNF-α. Blocking TLR9 had a negative effect (*P* = 0.03) on IL-17 production induced by *C. gattii* (Figure 5 for the effect on IL-1β and IL-17).

We performed these experiments also with *C. neoformans* var *grubii* (H99). The latter isolate did not elicit a substantial pro-inflammatory cytokine response in PBMCs, as shown in previous experiments with other strains. Moreover, we did not observe an increase in IL-1β and TNF-α production induced by *C. neoformans* var *grubii* when blocking TLR9 (results not shown).

## Discussion

In the present study we investigated the *in-vitro* cytokine production of human PBMCs incubated with 40 different heat-killed isolates of the *Cryptococcu*s *neoformans* species complex. We demonstrate that *C. gattii* isolates induces higher concentrations of pro-inflammatory and Th17/22 cytokines compared to *C. neoformans* var. *neoformans* and *C. neoformans* var. *grubii*. In addition, we found that clinical *C. gattii* isolates were able to induce higher amounts of cytokines than environmental isolates or clinical *C. neoformans* isolates. Furthermore, we demonstrated a contribution of TLR4 and TLR9, but no role for TLR2, in the host's cytokine response to *C. gattii*.

Our results indicate that *Cryptococcus neoformans* species complex seems to induce mainly a IL-22 response, with surprisingly low IL-17 production. This argues against a Th17 response to cryptococcal infection as we hypothesized, but rather to an exclusively IL-22 producing subset of Th-cells. A candidate for this areTh22 cells. These cells have not been found in mice so far; therefore only studies with human cells can be used to determine the role of this Th subset in host defense against cryptococcal infections. The aryl hydrocarbon receptor is identified to mediate IL-22 production without mediating IL-17 and seems to be critical in differentiation of naïve T cells to Th22 cells [16]. IL-22 is a unique cytokine in that it acts only on non-immune cells including keratinocytes, myofibroblasts and epithelial cells in tissues of the respiratory system, skin and digestive tract, which express receptors for this cytokine [17]. IL-22 promotes the production of antimicrobial agents called β-defensins by epithelial cells and serves in mucosal defenses against pathogens. It is tempting to speculate that IL-22 is important for the initial anti-cryptococcal defense because it has a function at the place of entrance of this yeast, namely the epithelial surface of the respiratory system. However, to confirm these speculations, further research should be attempted to identify the role of IL-22 and Th22 cells in clinical patients with cryptococcosis.

Higher amounts of the pro-inflammatory cytokines IL-1β, TNFα, IL-6, IL-17 and IL-22 by human PBMCs were induced by *C. gattii* compared to both varieties of *C. neoformans*, indicating that certain (virulence) factors of *C. gattii* are responsible for a more pronounced inflammatory reaction. This finding is strengthened as the same trend was seen in the hybrids containing *C. gattii* as a partner of the mating pair. Therefore we suggest that an inheritable factor is responsible for the difference in cytokine production.

Our finding that *C. gattii* induces a more powerful pro-inflammatory response aimed at more efficient defense against the pathogen is supported by the work of Ngamskulrungroj et al [18]. The authors compared the pathogenesis of the two *Cryptococcus* species in mice using an inhalation model and they found that in naive mice, *C. gattii* grew significantly slower in blood than *C. neoformans*. Infection with *C. gattii* was restricted to the lungs, while *C. neoformans* dissimenated to the brain causing meningoencephalitis. When mice were infected intravenously with low inoculums of yeast, *C. neoformans* was more virulent than *C. gattii*. Apparently, in this murine model the host's peripheral immune cells are able to clear *C. gattii* infection more efficiently, probably by a more adequate cytokine response. However, in humans, *C. gattii* species seem to be more virulent, as they are able to cause disease in apparently immunocompetent hosts. Large-scale environmental colonization for *C. gattii* was found during the Vancouver Island outbreak, whereas only relatively few people developed overt disease [6]. It can be hypothesized that a specific defect in the innate immune system of affected hosts predisposes them to infection with *C. gattii*. Furthermore, other factors such as intracellular survival, outgrowth or dissemination may also be important for virulence of *C. gattii*, independent of the initial pro-inflammatory cytokine response [19]. In our experiments we used PBMCs of healthy individuals who are expected to have an adequate immune response to *C. gattii*. These cells reflect the second line of defense when the yeast enters the host after inhalation. Our results showed a less optimal recognition and initial cytokine induction of *C. neoformans* var. *grubii* and var. *neoformans*, which suggests that in a host with inadequate cellular immunity this less optimal innate cytokine response leads more easily to infection with *C. neoformans* var. *grubii* and var. *neoformans* compared to *C. gattii*. Clinical data support this, since infections of immunocompromised hosts with *C. neoformans* var *grubii* is far more prevalent than infection with *C. gattii* [20].

A potential limitation of our study is that heat-killed instead of live cryptococci were used. However, at the temperatures used for heat-killing, most virulence factors (capsular polysaccharide, lipoproteins) are retained. Moreover, in number of previous studies, heat-killed cryptococci were used and significant inflammatory responses specific for capsulated and unencapsulated cryptococci were found [21], [22]. One study investigated lymphocyte proliferation after stimulation with live and heat-killed cryptococci and found no difference [23]. Thus, we feel that in this study, the use of heat-killed crytococci is justified.

Our experiments using a virulent *C. gattii* strain in stimulating PBMCs that were pre-incubated with specific PRR-blocking reagents indicate a role for TLR4 and TLR9 in recognizing *Cryptococcus* and subsequently modulation of the pro-inflammatory cytokine response. TLR4 seemed to be involved in mounting a pro-inflammatory cytokine response. Previous studies suggest that glucuronoxylomannan, the major capsular component [15] or other cryptococcal cell wall elements [24] are involved in binding to TLR4. In this study we did not design experiments in order to identify which cell wall components are involved in the initial cytokine response. Cytokine responses appeared to be independent of TLR2 recognition, since blocking of this receptor had no effect on cytokine concentrations. This contrasted with what is found in mice by Biondo *et al.* who demonstrated a key role of TLR2, but not of TLR4 [12]. Other studies, however, found no major role for TLR2 in survival of cryptococcal infections in a murine model [11], [13].

Based on our results, a special role in *Cryptococcus* recognition can be ascribed to TLR9. Unmethylated CpG-rich DNA is the best-known ligand for this receptor. Nakamura *et al.* have shown that TLR9 recognizes cryptococcal DNA [14]. We found that this receptor mediates IL-17 production, without any effect on IL-22. Conversely, blockade of TLR9 resulted in increased IL-1β production in response to *C. gattii*. The latter effect opposes the possible effect of TLR4. However, a specific combination of PRRs that bind available fungal PAMPs lead to pathways that interact with each other because of a limited set of shared adaptor molecules and transcription factors, and converge to a tailored response [25]. Likely, TLR9 and TLR4 work together in recognizing *Cryptococcus* and their signaling pathways interact downstream. Interestingly, we did not see TLR9 dependent negative modulation of *C. neoformans* var. *grubii*, indicating that the TLR9 dependent recognition of *Cryptococcus* is species-dependent. Negative modulation of immune responses to fungal pathogens mediated by TLR9 have been observed in other studies [26]. As the host's response to *C. gattii* relies on an initial pro-inflammatory cytokine response more than in *C. neoformans* infections, it can be speculated that susceptibility to *C. gattii* is influenced by subtle TLR polymorphisms and not necessarily by a defective adaptive immune response.

In the present study we investigated the *in-vitro* cytokine production of human PBMCs incubated with 40 different heat-killed isolates of *Cryptococcu*s *neoformans* species complex. We demonstrated that isolates of *C. gattii* induce higher concentrations of the pro-inflammatory cytokines IL-1β, TNF-α and IL-6 and the Th17/22 cytokines IL-17 and IL-22 compared to *C. neoformans* var *neoformans* and *C. neoformans* var *grubii*. In addition, we found that clinical *C. gattii* isolates induced higher amounts of IL-1beta and IL-6 than environmental isolates. Furthermore, we demonstrated a likely contribution of TLR4 and TLR9, but no role for TLR2, in the host's cytokine response to *C. gattii*. In conclusion, clinical *C. gattii* isolates induced a more pronounced inflammatory cytokine response compared to other *Cryptococcus* species and non-clinical *C. gattii* that is dependent on TLR4 and TLR9 as cellular receptors.

## Materials and Methods

### Cryptococcal strains

Forty cryptococcal isolates from the CBS Fungal Biodiversity Centre (Utrecht, the Netherlands) were used in this study. These isolates were obtained from laboratory, clinical, environmental and veterinary sources. A detailed overview of the origin, sero- and AFLP genotype of these isolates is provided in Table 1. Twenty-three isolates were identified as *C. gattii,* 5 *C. neoformans* var. *neoformans*, 5 *C. neoformans* var. *grubii* and 7 hybrids, 3 of which were interspecies hybrids between *C. gattii* and *C. neoformans* var. *neoformans* and 4 hybrids between both *C. neoformans* varieties. In addition, 11 Cuban isolates were used in separate experiments, all identified as *C. neoformans* var *grubii* (Table 2).

**Table 1:** Details of the 40 cryptococcal isolates.

No. in experiment	Isolate	Other specification	Species and varieties	Sero-type	AFLP-genotype	Origin	Reference/Source
1	125.91	CBS10512	*C. neoformans* var. *grubii*	A	1	Cryptococcal meningitis patient, Tanzania	Lengeler *et al.*, 2002
2	CBS8336		*C. neoformans* var. *grubii*	A	1	Decaying wood of *Cassia* tree, Brazil	Boekhout *et al.*, 2001
3	CBS8710	CBS10515, H99	*C. neoformans* var. *grubii*	A	1	Subculture of type strain of *Cryptococcus neoformans* var. *grubii* (H99)	Boekhout *et al.*, 2001
4	CBS996^(T)^		*C. neoformans* var. *grubii*	A	1	Clinical isolate, Argentina	Boekhout *et al.*, 1997
5	P152		*C. neoformans* var. *grubii*	A	1	AIDS patient, Zimbabwe	Boekhout *et al.*, 2001
6	B-3501	CBS6900	*C. neoformans* var. *neoformans*	D	2	Genetic offspring of CBS6885×CBS7000 ( = NIH12×NIH433)	Boekhout *et al.*, 2001
7	JEC20	CBS10511, NIH-B4476	*C. neoformans* var. *neoformans*	D	2	Congenic pair with JEC21 that differs only in mating type	Kwon-Chung *et al.*, 1992a
8	JEC21	CBS10513, NIH-B4500	*C. neoformans* var. *neoformans*	D	2	Congenic pair with JEC20 that differs only in mating type	Kwon-Chung *et al.*, 1992a
9	WM629^(R)^	CBS10079	*C. neoformans* var. *neoformans*	D	2	HIV positive human, reference strain of molecular type VNIV, Melbourne, Australia.	Meyer *et al.*, 1999
10	CBS6998	NIH365	*C. gattii* VGI	B	4	Human, Thailand	Boekhout *et al.*, 1997
11	CBS8273	CBS6289, RV20186, NIH-B-3939	*C. gattii* VGI	B	4	Subculture of type strain of *Cryptococcus gattii* (RV 20186)	Boekhout *et al.*, 1997
12	WM179^(R)^	CBS10078	*C. gattii* VGI	B	4	Immunocompetent human, reference strain of molecular type VGI, Sydney, Australia	Meyer *et al.*, 2003
13	WM276	CBS10510	*C. gattii* VGI	B	*4*	*Eucalyptus tereticornis*, Mt. Annan, New South Wales, Australia	Kidd *et al.*, 2005
14	CN043		*C. gattii* VGIII	B	5	Human, Auckland, New Zealand	Katsu *et al.*, 2004
15	CBS8755	HOO58-I-682	*C. gattii* VGIII	C	5A	Detritus of almond tree, Colombia	Boekhout *et al.*, 2001
16	WM161^(R)^	CBS10081	*C. gattii* VGIII	B	5B	*Eucalyptus camaldulensis* wood from hollow, reference strain of molecular type VGIII, San Diego, USA	Meyer *et al.*, 2003
17	WM728		*C. gattii* VGIII	B	5B	*Eucalyptus* sp. debris from car park of zoo, San Diego, USA	Boekhout *et al.*, 2001
18	CBS6955^(T)^	NIH191, ATCC32608	*C. gattii* VGIII	C	5C	Human, type strain of *Cryptococcus bacillisporus*, California, USA	Boekhout *et al.*, 1997
19	CBS6993	NIH18	*C. gattii* AFLP5 = VGIII	C	5C	Human, California, USA	Boekhout *et al.*, 1997
20	A1M R265	CBS10514	*C. gattii* VGII	B	6	Immunocompetent male, Duncan, Vancouver Island, Canada	Kidd *et al*., 2004
21	A1M R368	A1M-R376	*C. gattii* VGII	B	6	Immunocompetent male, Victoria, Canada	Kidd *et al.*, 2004
22	CBS1930		*C. gattii* VGII	B	6	Sick goat, Aruba	Boekhout *et al.*, 1997
23	CBS6956	NIH444, ATCC32609	*C. gattii* VGII	B	6	Immunocompetent human, Seattle, USA,	Boekhout *et al.*, 1997
24	WM178^(R)^	IFM50894, CBS10082	*C. gattii* VGII	B	6	Immunocompetent human, lung, reference strain of molecular type VGII, Sydney, Australia	Meyer *et al.*, 2003
25	AV55	CBS10090	*C. gattii* VGII	B	6A	HIV-negative human, Greece	Hagen *et al.* 2012
26	AV54	CBS10089	*C. gattii* VGII	B	6B	HIV-negative human, Greece	Hagen *et al.* 2012
27	B5742		*C. gattii* VGIV	C	7	Human, Punjab, India	Katsu *et al.*, 2004
28	B5748		*C. gattii* VGIV	B	7	HIV positive patient, India	Diaz and Fell, 2005
29	M27055		*C. gattii* VGIV	C	7	Clinical, Johannesburg, South Africa	Latouche *et al.*, 2002
30	WM779^(R)^	IFM50896, CBS10101	*C. gattii* VGIV	C	7	Cheetah, reference strain of molecular type VGIV, Johannesburg, South Africa	Meyer *et al.*, 2003
31	CBS10488	AMC770616	*C. gattii* AFLP4×*C. neoformans* AFLP2	BD	8	HIV-negative human, The Netherlands	Bovers *et al*., 2006
32	CBS10489	AMC2010404	*C. gattii* AFLP4×*C. neoformans* AFLP2	BD	8	HIV-positive human, The Netherlands	Bovers *et al*., 2006
33	CBS10496	LSPQ#308	*C. gattii* AFLP4×*C. neoformans* AFLP1	BD	9	HIV-positive human, Canada, visited Mexico	Bovers *et al*., 2008
34	CBS132		*-*	AD	3	Type strain *C. neoformans*, peach, Italy	Boekhout *et al.*, 2001
35	NYJ40		-	AD	3	-	Boekhout *et al*., 2001
36	RV52733		-	AD	3	-	Boekhout *et al*., 2001
37	RV52755		-	AD	3	-	Boekhout *et al*., 2001
38	CBS5467		*C. neoformans* var. *neoformans*	D	2	Milk from mastitic cow, Switzerland	Boekhout *et al.*, 1997
39	IHEM14941 Slimy	RV 63979, IHEM14941, CBS11687	*C. gattii*	B	10	HIV- patient from Mexico, Spain	Hagen *et al.* 2012
40	IHEM14941 White	RV 63979, IHEM14941	*C. gattii*	B	10	HIV- patient from Mexico, Spain	Hagen *et al.* 2012

**Table 2:** Details of 11 additional *C. neoformans* var *grubii* isolates, arranged by Microsatellite Complex (MC) [29].

Number in experiment	Isolate	Other specification	Species	Serotype	MC	Origin	Reference/Source
I	37-07-17	Cuba 617-05	*C. neoformans* var *grubii*	A	MC1	Clinical	Illnait Zaragozi *et al.*, 2010
II	44-08-52	Cuba CA 1-5	*C. neoformans* var *grubii*	A	MC1	Environmental	Illnait Zaragozi *et al*., 2010
III	37-07-03	Cuba 24-2b	*C. neoformans* var *grubii*	A	MC1	Environmental	Illnait Zaragozi *et al*., 2010
IV	36-10-01	Cuba CH-2	*C. neoformans* var *grubii*	A	MC2	Environmental	Illnait Zaragozi *et al*., 2010
V	44-08-16	Cuba 569-06	*C. neoformans* var *grubii*	A	MC2	Clinical	Illnait Zaragozi *et al*., 2010
VI	36-09-16	Cuba 225-99	*C. neoformans* var *grubii*	A	MC2	Clinical	Illnait Zaragozi *et al*., 2010
VII	36-09-32	Cuba 227-01	*C. neoformans* va*r grubii*	A	MC3	Clinical	Illnait Zaragozi *et al*., 2010
VIII	36-09-57	Cuba 0119	*C. neoformans* var *grubii*	A	MC3	Clinical	Illnait Zaragozi *et al*., 2010
IX	36-10-46	Cuba 30-2D	*C. neoformans var grubii*	A	MC3	Environmental	Illnait Zaragozi *et al*., 2010
X	36-10-56	Cuba 315-01	*C. neoformans var grubii*	A	MC4	Clinical	Illnait Zaragozi *et al*., 2010
XI	36-09-53	Cuba 098	*C. neoformans var grubii*	A	MC6	Clinical	Illnait Zaragozi *et al*., 2010

Prior to the experiments, the strains were freshly grown on Sabouraud dextrose agar plates. A suspension of each strain was prepared in sterile phosphate buffered saline (PBS), heat-killed overnight at 56°C and quantified by spectrophotometry at a wavelength of 530 nm. The suspensions were checked for fungal and bacterial growth on a Sabouraud dextrose agar plate and a blood agar plate respectively. No growth was observed after 5 days. All strains were stored at 4°C until used.

### *Candida* strain

Heat-killed *Candida albicans* ATCC MYA-3573 (UC 820), a well described clinical isolate, suspended in sterile PBS, was used as a positive control.

### Reagents and antibodies

*Bartonella* LPS, a penta-acylated LPS which is an antagonist of TLR4-dependent signaling, was obtained as previously described [27]. An anti-TLR2 monoclonal antibody from eBioscience (San Diego, CA, USA) was used, and an irrelevant isotype-matched murine IgG1 κ isotype (Biolegend, San Diego, CA, USA) as control. TLR9 inhibitory oligonucleotides ODN TTAGGG (anti TLR9) [28] and its negative control were obtained from InvivoGen (San Diego, CA, USA).

### Isolation and stimulation of PBMCs

Human peripheral blood mononuclear cells (PBMCs) were collected from buffy coats of healthy donors after written informed consent had been obtained. PBMCs were isolated using density gradient centrifugation on Ficoll-Hypaque (GE Healthcare, Uppsala, Sweden). The cells from the interphase were aspirated and washed three times in sterile PBS and resuspended in culture medium RPMI 1640 Dutch modification (Sigma-Alderich, St Louis, MO, USA) supplemented with 1% L-glutamine, 1% pyruvate and 1% gentamicin. Cells were counted in a Coulter Counter Z® (Beckman Coulter, Fullerton, CA, USA), and adjusted to 5×10^6^ cells/ml. Thereafter, they were incubated in a round-bottom 96-wells plate (volume 200 µl/well) at 37°C and 5% CO_2_ with either one of the heat-killed cryptococcal strains (final concentration of 10^7^/ml), or heat-killed *C. albicans* (final concentration of 10^5^/mL, which is known to induce substantial amounts of cytokines) or culture medium alone. After 24 hours or 7 days (in the presence of 10% human pool serum) supernatants were collected and stored at −20°C until being assayed.

In a subsequent experiment, PBMCs were preincubated for one hour with inhibitory ligand for TLR4 (*Bartonella quintana* LPS (200 ng/ml) or culture medium as control, anti-TLR2 or control antibody (10 µg/ml), TLR9 inhibitory oligonucleotides and its negative control (25 µg/ml). After preincubation, *C. gattii* B5742, isolate 27 in the previous experiment, or specific TLR ligands were added, such as Pam3cys or E.coli LPS (10 µg/ml and 10 ng/ml respectively] and PBMCs were incubated as described.

### Cytokine assays

Tumor necrosis factor-α (TNF-α), Interleukin-1β (IL-1β), IL-6 and IL-1 receptor antagonist (IL-1Ra) concentrations were determined from the culture supernatant after 24 hours of incubation using commercially available ELISA kits (TNF-α, IL-1β and IL-1Ra: R&D systems, Minneapolis, MN, USA. IL-6: Sanquin,Amsterdam, the Netherlands) according to the manufacturer's instructions. T-cell derived cytokines IL-17 and IL-22 concentrations were determined in the supernatant after 7 days of incubation using ELISA kits (R&D systems). Lower detection limits were 78 pg/ml, 39 pg/ml, 15 pg/ml, 200 pg/ml, 40 pg/ml and 78 pg/ml for TNF-α, IL-1β, IL-6, IL-1Ra, IL-17 and IL-22 respectively.

### Ethics statement

Written informed consent of healthy donors was provided. The study was approved by the Medical Ethical Committee Arnhem-Nijmegen in the Netherlands.

### Statistical analysis

Results from at least three different experiments with a range of 5–7 donors were pooled and analyzed using GraphPad Prism 5 software (GraphPad, San Diego, CA). Data are given as mean ± SE. The Mann-Whitney *U*-test for unpaired, nonparametrical data was used to compare differences in cytokine production between two groups. The Kruskal-Wallis test with Dunn's multiple comparison test was used when more than two groups were compared. The Wilcoxon matched-pairs signed rank test was used to analyze differences in cytokine production between inhibitors and their controls in the inhibition experiments. The level of significance was set at p<0.05.

## Supporting Information

Figure S1 **IL-1β induction by Pam3cys and** ***E. coli*** **LPS after blocking of TLR2 and TLR4 respectively.** IL-1β production by human PBMCs is shown (A) induced by pam3cys [10 µg/ml] after preincubated for one hour with anti-TLR2 or control antibody [10 µg/ml] and (B) by *E. coli* LPS [10 ng/ml] after preincubation for one hour with TLR4 antagonist *Bartonella quintana* LPS [200 ng/ml] or culture medium. Mean values (n = 10) ± SE of five independent experiments are presented.
(TIF)
