# Supplementary figures and images for: Cryptococcus gattii Induces a Cytokine Pattern That Is Distinct from Other Cryptococcal Species
Source: PLoS One. 2013 Jan 31;8(1):e55579. doi: 10.1371/journal.pone.0055579 (PMC3561320; doi:10.1371/journal.pone.0055579)

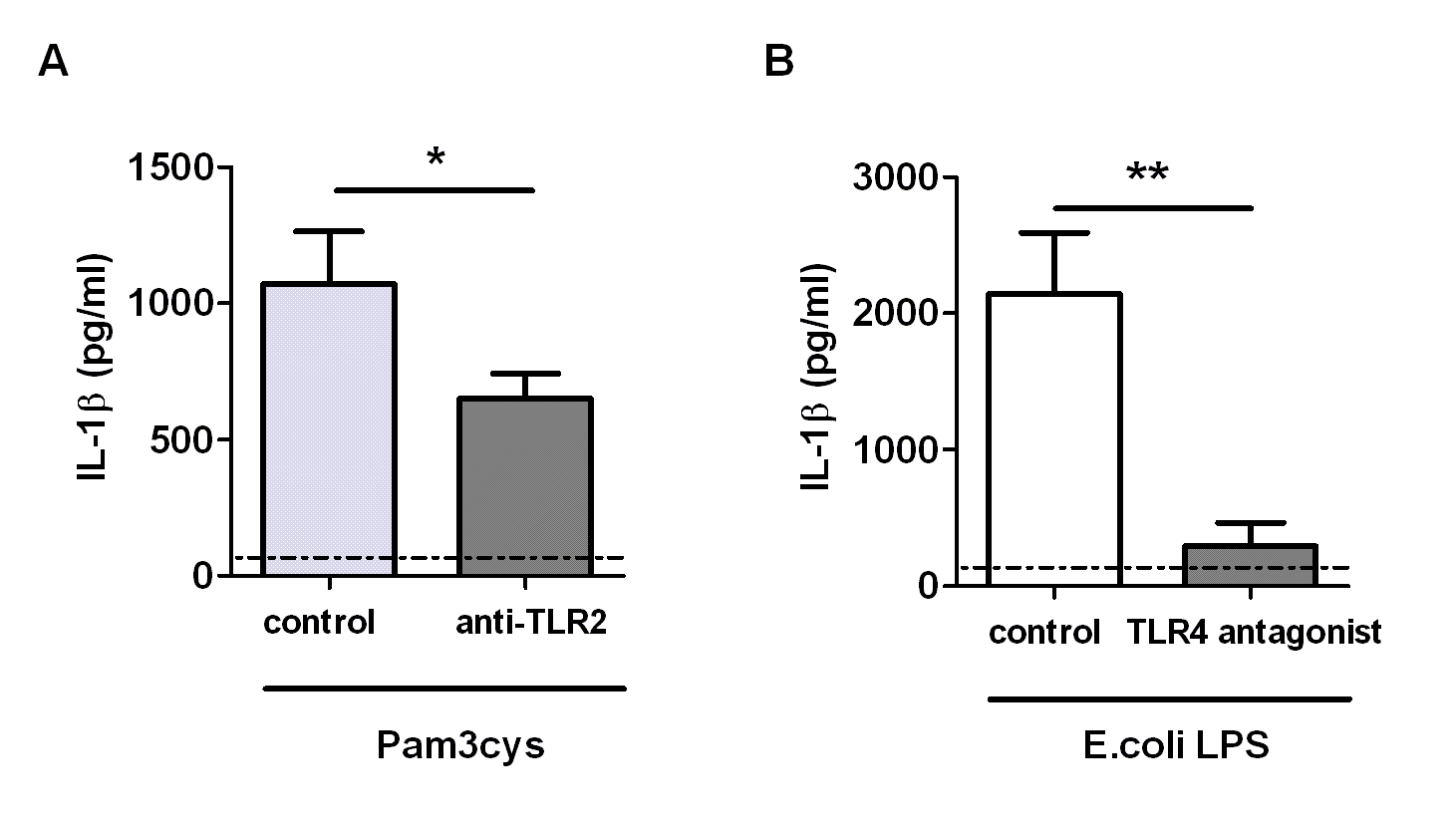

Supplement: Figure S1 — IL-1β induction by Pam3cys and E. coli LPS after blocking of TLR2 and TLR4 respectively. IL-1β production by human PBMCs is shown (A) induced by pam3cys [10 µg/ml] after preincubated for one hour with anti-TLR2 or control antibody [10 µg/ml] and (B) by E. coli LPS [10 ng/ml] after preincubation for one hour with TLR4 antagonist Bartonella quintana LPS [200 ng/ml] or culture medium. Mean values (n = 10) ± SE of five independent experiments are presented. (TIF) [file pone.0055579.s001.tif]
